# Dissecting the multifaceted impact of statin use on fatty liver disease: A multidimensional study

**DOI:** 10.1016/j.ebiom.2022.104392

**Published:** 2022-12-08

**Authors:** Ibrahim Ayada, Laurens A. van Kleef, Huai Zhang, Kuan Liu, Pengfei Li, Yasir J. Abozaid, Marla Lavrijsen, Harry L.A. Janssen, Luc J.W. van der Laan, Mohsen Ghanbari, Maikel P. Peppelenbosch, Ming-Hua Zheng, Robert J. de Knegt, Qiuwei Pan

**Affiliations:** a Department of Gastroenterology and Hepatology, Erasmus MC-University Medical Center, Rotterdam, the Netherlands; b Department of Biostatistics and Records Room, Medical Quality Management Office, the First Affiliated Hospital of Wenzhou Medical University, Wenzhou, China; c Department of Surgery, Erasmus MC-University Medical Center, Rotterdam, the Netherlands; d Department of Epidemiology, Erasmus MC-University Medical Center, Rotterdam, the Netherlands; e Toronto Center for Liver Disease, Toronto General Hospital, University of Toronto, Canada; f NAFLD Research Center, Department of Hepatology, the First Affiliated Hospital of Wenzhou Medical University, Wenzhou, China; g Institute of Hepatology, Wenzhou Medical University, Wenzhou, China; h Key Laboratory of Diagnosis and Treatment for The Development of Chronic Liver Disease in Zhejiang Province, Wenzhou, China

**Keywords:** NAFLD treatment, Statins, General and patient population, Meta-analysis, Organoids, Metabolic dysfunction, FLI, fatty liver index, MAFLD, metabolic dysfunction associated fatty liver disease, NAFLD, non-alcoholic fatty liver disease, NASH, non-alcoholic steatohepatitis, qPCR, quantitative polymerase chain reaction

## Abstract

**Background:**

Statin use could benefit patients with non-alcoholic fatty liver disease (NAFLD), but the evidence is segmented and inconclusive. This multidimensional study comprehensively investigated the potential benefits and mechanism-of-action of statins in NAFLD.

**Methods:**

A cross-sectional investigation was performed within the Rotterdam Study (general population; n = 4.576) and the PERSONS cohort (biopsy-proven NAFLD patients; n = 569). Exclusion criteria were secondary causes for steatosis and insufficient data on alcohol, dyslipidemia or statin use. Associations of statin use with NAFLD (among entire general population), fibrosis and NASH (among NAFLD individuals and patients) were quantified. These results were pooled with available literature in meta-analysis. Last, we assessed statins’ anti-lipid and anti-inflammatory effects in 3D cultured human liver organoids and THP-1 macrophages, respectively.

**Findings:**

Statin use was inversely associated with NAFLD in the Rotterdam study compared to participants with untreated dyslipidemia. In the PERSONS cohort, statin use was inversely associated with NASH, but not with fibrosis. The meta-analysis included 7 studies and indicated a not significant inverse association for statin use with NAFLD (pooled-Odds Ratio: 0.69, 95% Confidence Interval: 0.46–1.01) and significant inverse associations with NASH (pooled-OR: 0.59, 95% CI: 0.44–0.79) and fibrosis (pooled-OR: 0.48, 95% CI: 0.33–0.70). In vitro, statins significantly reduced lipid droplet accumulation in human liver organoids and downregulated expression of pro-inflammatory cytokines in macrophages.

**Interpretation:**

Pooled results demonstrated that statin use was associated with a lower prevalence of NASH and fibrosis and might prevent NAFLD. This may be partially attributed to the anti-lipid and anti-inflammatory characteristics of statins. Given their under-prescription, adequate prescription of statins may limit the disease burden of NAFLD.

**Funding:**

ZonMw, KWF, NWO, SLO, DGXII, RIDE, National and regional government, Erasmus MC and Erasmus University.

**Research in context**

**Evidence before this study**

NAFLD has become the most prevalent chronic liver disease and the need for effective pharmaceutical treatment is unmet. We searched PubMed, Web of Science, Cochrane register, Embase from database inception until January 2022 using keywords of “NAFLD” AND “statins” or affiliated terms. Studies from any country and in any language were assessed. Clinical and experimental studies have indicated that statins may be beneficial in preventing NAFLD, NASH and fibrosis. Interestingly, larger effects have been demonstrated for higher dosage and longer duration of statin use, supporting the potential benefits of statin treatment. However, controversy remains, as large clinical trials are scarce and the available evidence is segmented, inconclusive, and contradictory. Hence, we designed a multi-dimensional study to investigate the potential benefits and multi-faceted impact of statins on liver health.

**Added value of this study**

This study comprehensively investigated the hepatoprotective effects of statins in the general population, in biopsy proven NAFLD patients and through meta-analysis as well as experimental studies, aiming to settle the ongoing discussion. We now provide evidence regarding the hepatoprotective effects of statins on NAFLD, NASH, and fibrosis by pooling available and newly presented data. These associations may be explained by anti-inflammatory and direct anti-steatosis effects as shown in our experimental models.

**Implications of all the available evidence**

Statins may lower the risk of NAFLD and should therefore be considered among those at risk of NAFLD development. As there is under-prescription of statins in NAFLD patients, there is a lot to gain by just prescribing statins according to guidelines. Moreover, statins should be considered in patients with NAFLD, as statins were effective in reducing the risk of NASH and fibrosis. Since statins are safe and inexpensive, it is striking that currently 40–50% of patients with NAFLD do not receive treatment while indicated. Therefore, increased awareness among physicians involved in the multidisciplinary treatment of NAFLD is required. Further studies should investigate whether statins are hepatoprotective among those without a conventional indication for statin treatment.

## Introduction

Non-alcoholic fatty liver disease (NAFLD) is a major health concern with an estimated prevalence exceeding 25% globally, driven by an alarming increase in obesity and metabolic disorders.^1^ Despite major efforts, there is still an urgent need for effective treatment since novel pharmaceutical agents did not meet the required endpoints yet.

Interestingly, some studies have suggested that statins (HMG-COA reductase inhibitors) might effectively reduce the risk of NAFLD.^2^^,^^3^ Experimental and clinical studies have shown that the effects of statins go beyond their cardiovascular-protective ability and consist of anti-inflammatory, anti-thrombotic and anti-fibrotic properties and may thus inhibit progression from simple steatosis to fibrosis and non-alcoholic steatohepatitis (NASH).^4^, ^5^, ^6^ Moreover, in a randomized controlled trial, statins lowered portal hypertension in patients with advanced disease such as cirrhosis.^7^

On the contrary, prescribing statins to patients with chronic liver disease often raises the issue of hepatotoxicity among clinicians given that statins are metabolized in the liver by CYP450 isoenzymes.^8^ However, one of the most common side effects of statins, asymptomatic transaminitis, is still relatively uncommon (around 3%), occurs in the first year of treatment initiation, is dose-dependent, and is usually self-limiting.^8^ Moreover, statin use was safe even among those with NAFLD and elevated liver enzymes, meaning that statins might target both genesis or deterioration of NAFLD and risk of coronary artery disease, which is increased in NAFLD patients.^9^ Lastly, a meta-analysis showed that the prevalence of transaminitis among patients using statins or other lipid lowering medication is not significantly different from that of individuals using placebo.^10^^,^^11^

Convincing evidence regarding the safety of statins in NAFLD patients is available, but the evidence concerning their hepatoprotective effects is segmented and inconclusive.^12^^,^^13^ Considering the complexity of addressing this question, this study took a multidimensional approach. We first comprehensively assessed the associations of statin use with NAFLD, NASH and fibrosis in a large general population cohort, a biopsy-proven NAFLD patient cohort, and a meta-analysis of pooling existing data. Finally, we experimentally tested the effects of statins on lipid accumulation and inflammatory gene expression in human liver organoids and macrophages, respectively, to explore potential mechanism-of-action.

## Methods

### Study design and procedures

To assess the potential multifaceted effects of statins on the NAFLD disease spectrum, we performed a multidimensional study comprising a cross-sectional investigation in a general population cohort and a NAFLD patient cohort, a meta-analysis, and finally an experimental exploration.

The Rotterdam Study is an ongoing population-based cohort.^14^ Participants visiting the research center between 2009 and 2014 were eligible for inclusion (n = 5.967). Exclusion criteria were secondary causes for steatosis (n = 922 [excessive alcohol consumption, viral hepatitis and steatogenic drug use]) or insufficient data (n = 469) on alcohol consumption, dyslipidemia or statin use (Supplementary Table S1). NAFLD was defined as steatosis on abdominal ultrasound performed by a single sonographer as hyperechoic liver parenchyma compared to kidney or spleen. Liver stiffness data was available for 72% of this cohort and fibrosis was defined as liver stiffness ≥8.0 kPa after discarding unreliable measurements according to the Boursier criteria (n = 141) and measurements among those with heart failure (n = 70).^15^ Data regarding statin use was obtained by linkage with the participants' pharmacies and verified during an interview. Dyslipidemia was defined as either hypo-HDL or hypertriglyceridemia, applying cut-offs from the Adult Treatment Panel III criteria for the metabolic syndrome.^16^

In order to prevent distortion of those without dyslipidemia who are unlikely to benefit from statin use, we identified three subgroups based on statin use and dyslipidemia: statin use, non-treated dyslipidemia and no dyslipidemia. These groups were used to quantify the association of statin use with NAFLD by logistic regression. Subsequently, we selected participants with NAFLD to further investigate the potential benefits of statins on fibrosis. For this analysis, patients with NAFLD using statins were compared to NAFLD patients without statin use, regardless of dyslipidemia. Multivariable models were adjusted for age, sex, hypertension, (pre)diabetes, and high waist circumference. For sensitivity analyses, NAFLD was replaced by the newly introduced definition of MAFLD,^17^^,^^18^ the cut-off for fibrosis was lowered to 7.0 kPa to increase statistical power, and BMI was added to the final model.

The PERSONS (Prospective Epidemic Research Specifically Of NASH) cohort is a well-characterized cohort of biopsy-proven Chinese NAFLD patients, who visited the First Affiliated Hospital of Wenzhou Medical University between 2016 and 2019.^19^^,^^20^ Patients with NAFLD and available metabolic health data who required liver biopsy due to abnormal liver imaging, abnormal liver function test, and/or abnormal fibrosis tests, were eligible for inclusion (n = 569). NASH was defined as the simultaneous presence of steatosis, lobular inflammation, and hepatocellular ballooning.^21^ Fibrosis was defined as Brunt classification ≥ F2.^22^ Data on statin use was obtained from the patient's healthcare system. Associations for statin use with NASH and fibrosis were quantified with logistic regression using the same multivariable models.

A meta-analysis was performed according to the PRISMA guidelines in order to comprehensively assess the potential associations of statins with NAFLD, NASH and fibrosis. The systematic literature search was performed on 10th of January 2022 in Medline, Embase and Web of Science, using NAFLD and statins with affiliated terms (detailed search and screening methods are included in Supplementary Methods and the full search strategy in Supplementary Table S2). Briefly, we included original studies that reported on the association between statin use and our primary outcomes in individuals with metabolic dysfunction. Primary outcomes were the presence of (1) NAFLD, (2) NASH, or (3) fibrosis. In addition, findings from the Rotterdam Study and PERSONS cohort in the current study, were also included. Odds ratios were extracted from fully adjusted models and pooled using generic inverse variance and random-effects models. Furthermore, we assessed excessive influence of our results and other individual studies on the pooled outcome by excluding one study at a time.

To assess the direct effects on lipid accumulation and to identify potential causal pathways, we tested simvastatin and lovastatin in 3D cultured primary human liver organoids. Organoids were cultured in matrigel with Advanced DMEM/F12 (Life Technologies, cat. no. 12634-010), in addition to 1 M HEPEs (Lonza, cat. no. 17-737E), ultraglutamine (Lonza, cat. no. BE17-605E/U1) and penicillin-streptomycin as the basic culture medium, supplied with 1:50 B27 supplement (minus vitamin A), 1:100 N2 supplement, 1 mM N-acetylcysteine, 10 mM nicotinamide, 50 ng/ml EGF, 100 ng/ml FGF-10, 50 ng/ml HGF, 5 μM A83-01, 10 μM forskolin, 10 nM gastrin and 10% R-spondin1 (produced by 293T-H-RspoI-Fc cell line). The organoids were cultured for approximately one week in which the medium was refreshed every 72 h. The use of human liver tissues for research purposes was approved by the Medical Ethical Council of Erasmus MC and informed consent was given (MEC-2014-060).

After induction of lipid droplet accumulation for 96 h using sodium lactate, sodium pyruvate and octanoic acid, lipids and nuclei were then subsequently stained with AdipoRed (Lonza, cat. no. PT-7009) and Hoechst 33342 (Life Technologies, cat. no. H3570). Detailed experimental methods related to liver organoids-based fatty liver disease model are described in Supplementary Methods and in our previous study of establishing this model.^23^ Moreover, pathological inflammation is a crucial driver of NAFLD progression towards NASH, which is mainly mediated by macrophages.^24^ Therefore, we performed qpCR to test the effects of simvastatin and lovastatin on the expression of inflammatory genes in cultured human THP-1 macrophages. Briefly, THP-1 cell line was cultured in RPMI 1640 medium (ThermoFisherScientific, Waltham, MA, USA), complemented with 10% (v/v) inactivated fetal bovine serum (FBS) with penicillin-streptomycin. For differentiation into macrophages, THP-1 cells were treated with 20 ng/mL of phorbol 12-myristate 13-acetate (PMA) at 37 °C for 48 h. Lastly, by using human liver organoids, we tested the effects of statin treatment on expression of genes involved in steatosis, fat metabolism, and mitochondrial function and morphology. Detailed experimental methods were described in Supplementary Methods.

Authentication of cell lines was performed using the short tandem repeat genotyping assay at the Molecular Diagnostics Department, Erasmus Medical Center. The mycoplasma-free status was regularly (commercially) checked and confirmed based on the real-time PCR method at Eurofins GATC-Biotech (Konstanz, Germany).

### Statistics

Statistical analyses were performed in R version 4.0.4 (The R foundation for statistical computing, Vienna, Austria), SPSS v26.0 (IBM Corp., Armonk, NY) and GraphPad Prism (version 8.0.2; GraphPad Software Inc., La Jolla, CA). In both cohorts, missing data was <0.25% and therefore not imputed. P-values of <0.05 were considered statistically significant. The meta-analysis was performed using R-package *meta* version 4.18-2 and *metafor* version 3.0-2.

### Ethics

The Rotterdam Study has been approved by the Medical Ethics Committee of the Erasmus MC (MEC-02.1015) and by the Dutch Ministry of Health, Welfare and Sport (1071272-159521-PG) and has been entered into the WHO International Clinical Trials Registry Platform (NTR6831). The PERSONS cohort study was approved by the internal review board for ethics at the First Affiliated Hospital of Wenzhou Medical University (2016-246, 1 December 2016) and was registered in the Chinese Clinical Trial Registry (ChiCTR-EOC-17013562). All participants signed a written informed consent to participate in this study.

### Role of funders

The funders of the study did not participate in study design, data collection, data analysis, data interpretation, or writing of the manuscript.

## Results

We analyzed 4.576 participants from the Rotterdam Study (age: 69.9 ± 9.2 year; male 41.0%, BMI 27.6 ± 4.3 kg/m^2^), of whom 1.591 (34.8%) had NAFLD. Among participants with NAFLD, valid liver stiffness measurement was available in 65.7% (n = 1.046), of whom 9.7% (n = 101) had fibrosis. In this cohort, 28.4% (n = 1.298) used statins, 24.3% (n = 1.110) had non-treated dyslipidemia and 47.4% (n = 2.168) had no dyslipidemia. Of note, statin users had more metabolic comorbidity (e.g. diabetes [33% vs 14%] and hypertension [90% vs 74%]) compared to the non-treated dyslipidemia group. As expected, HDL (1.4 vs 1.2 mmol/L) and triglycerides (1.4 vs 1.9 mmol/L) levels were favorable in those on statin treatment (Table 1).

**Table 1:** Participants’ characteristics.

	Rotterdam study			PERSONS cohort	
	Statin use n = 1.298	Untreated dyslipidemia n = 1.110	No dyslipidemia n = 2.168	Statin use n = 73	No statin use n = 496
**Demographics**					
Age (years)	71.9 (8.2)	69.0 (9.4)	69.2 (9.4)	47.9 (12.2)	41.5 (12.1)
Male	645 (49.7)	432 (38.9)	800 (36.9)	50 (68.5)	361 (72.8)
**Metabolic health**					
BMI (kg/m^2^)	28.4 (4.2)	28.6 (4.6)	26.6 (4.1)	26.6 (3.1)	26.6 (3.4)
High waist circumference	682 (52.6)	612 (55.2)	721 (33.3)	53 (72.6)	342 (69.0)
Diabetes	418 (33.0)	153 (14.0)	146 (6.9)	45 (61.6)	100 (20.2)
Hypertension	1163 (89.6)	819 (73.8)	1386 (63.9)	37 (50.7)	97 (19.6)
**Biochemistry**					
AST (U/L)	25 [21, 30]	24 [21, 28]	24 [21, 27]	31 [24, 45]	34 [25, 54]
ALT (U/L)	20 [16, 26]	19 [15, 26]	17 [14, 22]	40 [26, 62]	52 [29, 92]
Trigelycerides (mmol/L)	1.4 [1.0, 1.9]	1.9 [1.5, 2.2]	1.1 [0.9, 1.3]	1.9 [1.3, 2.7]	1.9[1.4, 2.4]
HDL (mmol/L)	1.4 (0.4)	1.2 (0.3)	1.7 (0.4)	1.03 (0.24)	1.02 (0.25)
**Liver assessment**					
NAFLD	554 (42.7)	524 (47.2)	513 (23.7)	73 (100)	496 (100)
NASH	–	–	–	41 (56.2)	351 (70.8)
Fibrosis^a^	54 (6.5)	42 (5.5)	84 (5.1)	14 (19.2)	91 (18.3)

Data is presented as mean (SD), median [P25–P75] or n and percentage.

Abbreviations: ALT, alanine aminotransferase; AST, aspartate aminotransferase; BMI, body mass index; HDL, high density lipoprotein; NAFLD, non-alcoholic fatty liver disease; NASH, on-alcoholic steatohepatitis.

a Based on LSM ≥8.0 kPa (in Rotterdam Study) or biopsy (in PERSONS cohort).

Importantly, statin treatment was inversely associated with NAFLD (OR: 0.72, 95% CI: 0.59–0.86; Table 2) compared to participants with non-treated dyslipidemia, adjusted for age, sex, hypertension, (pre)diabetes, and high waist circumference. However, participants without dyslipidemia (and not on statin therapy) had the lowest odds of NAFLD (OR: 0.50, 95% CI: 0.42–0.60). The association between statin use and fatty liver disease was consistent if NAFLD was replaced by MAFLD (Table 2). Among participants with NAFLD and reliable liver stiffness measurement (n = 1.046), statins were used in 32% and was not significantly associated with fibrosis (OR: 0.65, 95% CI: 0.40–1.07), in fully adjusted models. However, in a sensitivity analysis, the statistical power increased by using a more lenient definition of fibrosis (liver stiffness ≥7.0 instead of 8.0 kPa), and in this analysis statin use was significantly associated with lower prevalence of fibrosis (OR: 0.54, 95% CI: 0.36–0.82; Table 2). After additional adjusting for BMI, similar results were obtained for the associations between statin use and NAFLD or fibrosis.

**Table 2:** Association for statin use with NAFLD, NASH and fibrosis in fully adjusted models.

Rotterdam study				
	n	OR	95% CI	P
**NAFLD**				
Untreated dyslipidemia	1110	Reference group		
Statin treatment	1298	0.72	0.60–0.86	<0.001
No dyslipidemia	2168	0.50	0.42–0.60	<0.001
**MAFLD**				
Untreated dyslipidemia	1389	Reference group		
Statin treatment	1665	0.73	0.62–0.87	<0.001
No dyslipidemia	2836	0.48	0.41–0.56	<0.001
**Liver stiffness ≥ 8.0 kPa**				
No statin treatment	710	Reference group		
Statin treatment	334	0.65	0.40–1.07	0.096
**Liver stiffness ≥ 7.0 kPa**				
No statin treatment	710	Reference group		
Statin treatment	334	0.54	0.36–0.82	0.004

PERSONS Cohort				
	n	OR	95% CI	P
**NASH**				
No statin treatment	496	Reference group		
Statin treatment	73	0.55	0.32–0.95	0.031
**Fibrosis**				
No statin treatment	496	Reference group		
Statin treatment	73	0.86	0.44–1.68	0.857

Results were obtained with logistic regression and adjusted for age, sex, hypertension, (pre)diabetes, and high waist circumference. The reference group for NAFLD and MAFLD analysis were participants with untreated dyslipidemia. NASH and fibrosis analyses were performed among individuals with NAFLD and reference group was no statin use.

Next, we analyzed 569 patients with biopsy-proven NAFLD from the PERSONS cohort (age: 42.3 ± 12.3 year; male 72.2%; BMI: 26.6 ± 3.3 kg/m^2^), of whom 68.9% (n = 392) had NASH, and 18.5% (n = 105) fibrosis. Additional baseline characteristics are shown in Table 1. In this cohort 12.8% (n = 73) used statins, which was inversely associated with NASH (OR: 0.55, 95% CI: 0.32–0.95) but not with fibrosis (OR: 0.86, 95% CI: 0.44–1.68) in fully adjusted models (Table 2).

**Fig. 1:**
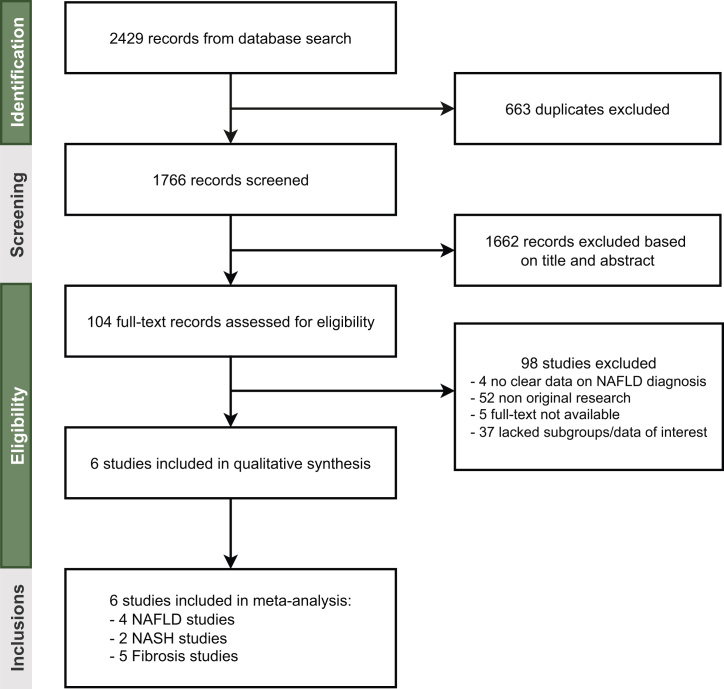
**Selection of studies**. PRISMA flowchart of literature search and study identification.

**Fig. 2:**
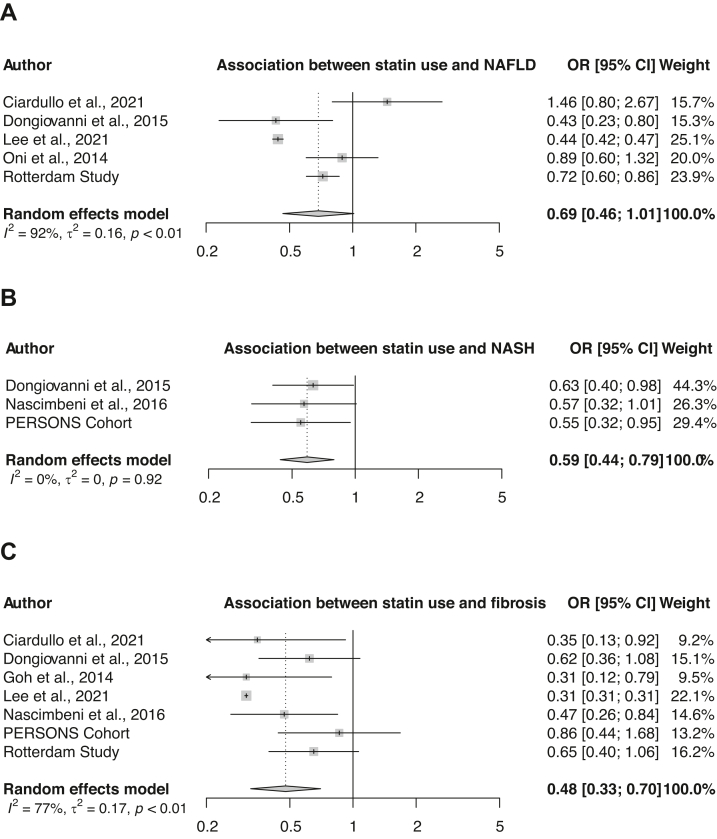
**Forest plots showing the associations between statin use and the NAFLD disease spectrum.** Random effects models were used to assess pooled odds ratio of the association between statin use and NAFLD (A), statin use and NASH (B), and statin use and fibrosis(C).

To further validate observed trends and our findings and to increase statistical power, we performed a meta-analysis. Our systematic search found 1766 unique articles, of which 6 were included for the analysis (Fig. 1).^25^, ^26^, ^27^, ^28^, ^29^, ^30^ Combining the results obtained from our Rotterdam Study and PERSONS cohort, 5 studies were finally included for NAFLD, 3 for NASH and 7 for fibrosis analysis, respectively. Study characteristics, including the definitions of NAFLD, NASH and fibrosis, as well as quality assessment of included studies are shown in Supplementary Tables S3 and S4, respectively. Different multivariable models were used across the included studies, but all extracted odds ratios were accounted for age, sex and metabolic health (e.g. BMI, metabolic syndrome and/or diabetes). Pooled results indicated a not significant, but inverse association for statin use with steatosis (pooled OR: 0.69, 95% CI: 0.46–1.01; Fig. 2A), a significant inverse association with NASH (pooled OR: 0.59, 95% CI: 0.44–0.79; Fig. 2B) and a significant inverse association with fibrosis (pooled OR: 0.48, 95% CI: 0.33–0.70; Fig. 2C), among those with metabolic dysfunction. Excessive influence analysis did not reveal a study, nor our own results, with a specifically large influence on the pooled odds ratios. Interestingly, in this excessive influence analysis, when excluding for example the studies of Ciardullo et al.^25^ or Oni et al.,^27^ there was a significant inverse association for statin use with NAFLD (Supplementary Figure S1).

**Fig. 3:**
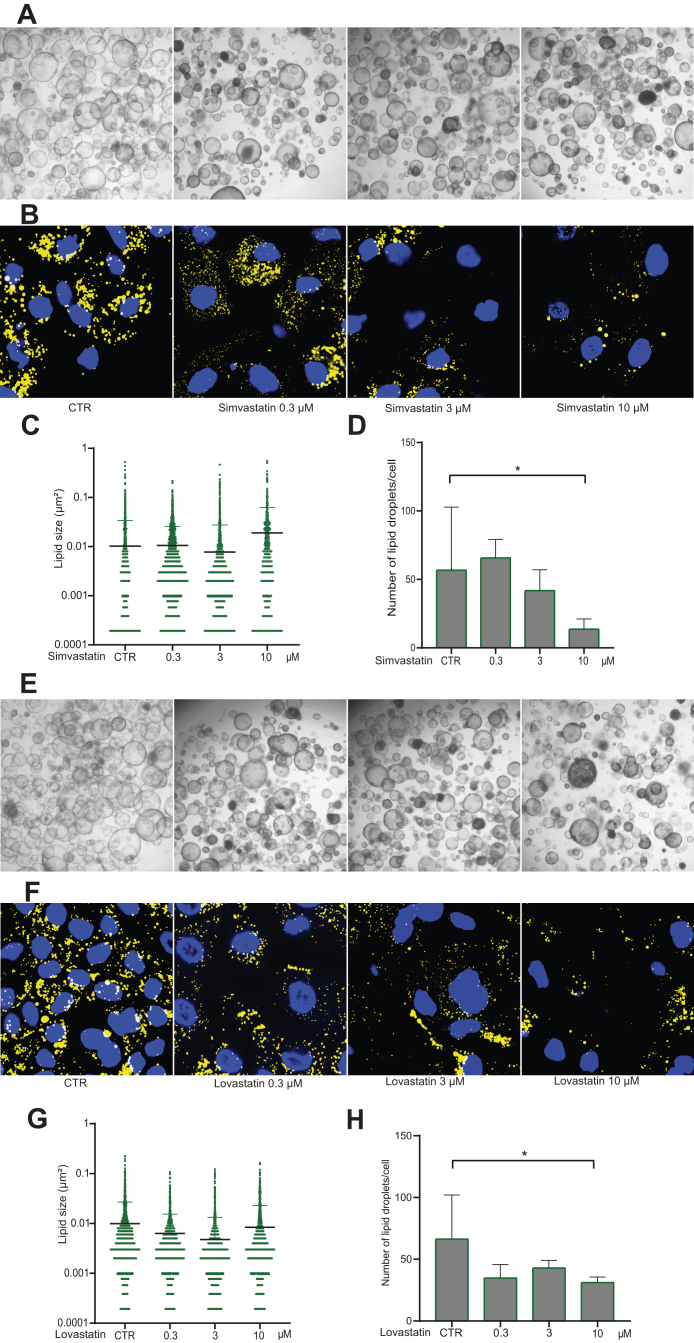
**Effects of simvastatin and lovastatin on lipid accumulation in 3D cultured human liver organoids.** (A) Bright field images representing morphology of organoids treated with LPO only (CTR) or with LPO and different concentrations of simvastatin (20× magnification). (B) Confocal images of lipid droplets (yellow, stained by AdipoRed) and nuclei (blue, stained by Hoechst) (2000× magnification at room temperature). (C) Effect of simvastatin on the size of the induced lipid droplets; 2565, 3301, 2902, 866 lipids were captured for CTR, 0.3 μM, 3 μM and 10 μM respectively. (D) Effect of simvastatin on the number of induced lipid droplets, normalized for the amount of cells (n = 4). (E) Bright field images representing morphology of organoids treated with LPO only (CTR) or with LPO and different concentrations of lovastatin (20× magnification). (F) Confocal images of lipid droplets (yellow, stained by AdipoRed) and nuclei (blue, stained by Hoechst) (2000× magnification at room temperature). (G) Effect of lovastatin on the size of induced lipid droplets; 1772, 1686, 2390, 1728 lipids were captured for CTR, 0.3 μM, 3 μM and 10 μM respectively. (H) Effect of lovastatin on the number of induced lipid droplets, normalized for the amount of cells (n = 4). Data are presented as mean ± SD, ∗P < 0.05.

**Fig. 4:**
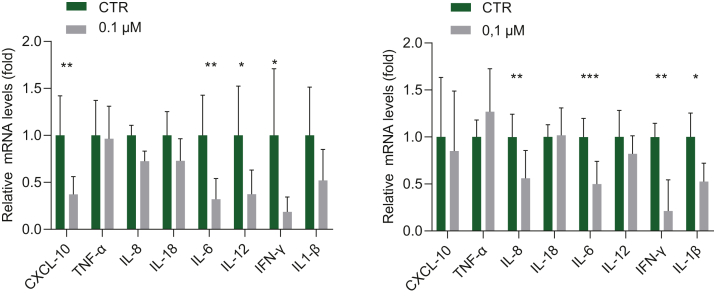
**The effect of statin treatment on inflammatory gene expression in human macrophages**. Human THP-1 monocytes were differentiated into macrophages and treated with statins. Relative gene expression is represented as CTR (untreated) and as treated with simvastatin 0.1 μM (left panel, n = 8) and lovastatin 0.1 μM (right panel, n = 6). Data are presented as mean ± SD, ∗P < 0.05, ∗∗P < 0.01, ∗∗∗P < 0.001.

To investigate whether statins have a direct effect on lipid accumulation, we tested simvastatin and lovastatin in our fatty liver model of primary human liver organoids mimicking steatosis.^23^ We found that experimental treatment with simvastatin and lovastatin appears to attenuate the intracellular accumulation of lipid droplets. Although the effect on lipid droplet size was mild, we observed significant reduction of the average number of formed lipid droplets per viable cell with more prominent effects at higher concentrations of statins (Fig. 3). Of note, there is no major effect of statin treatment on the viability of liver organoids (Supplementary Figure S2). Then, we tested the effects on inflammatory gene expression in human THP-1 macrophages since macrophage-driven inflammation is a driver of NAFLD disease progression. With a low concentration (0.1 μM) of simvastatin and lovastatin, significant inhibition on the expression of inflammatory genes such as CXCL10, IL-6, IL-12, and interferon-gamma was already observed (Fig. 4). Finally, we investigated the effects of statin treatment on the expression of genes involved in steatosis, fat metabolism and mitochondrial function and morphology. Interestingly we observed a clear trend of upregulating SREBP1, PNPLA3 and TMS6F after induction of lipid droplet accumulation, followed by an inhibition of these genes by both simvastatin and lovastatin (Supplementary Figure S3).

## Discussion

In this multidimensional study, we comprehensively investigated the potential benefits of statin use on liver health within the NAFLD disease spectrum. Our pooled results demonstrated that among those with metabolic dysfunction, the use of statins was significantly associated with a lower prevalence of NASH and fibrosis and may also prevent NAFLD. These multifaceted beneficial effects are likely attributed to multiple mechanism-of-actions.

In the Rotterdam Study, a well-defined population-based cohort, we found a beneficial association between statin use and NAFLD prevalence compared to participants with untreated dyslipidemia. However, participants without dyslipidemia (and without statin use) were even at lower risk of NAFLD, indicating that statins could not entirely normalize the odds for NAFLD. Similar results were obtained when NAFLD was replaced by MAFLD, indicating that the beneficial effect of statins can be extrapolated to the larger MAFLD population. This anti-steatotic property is supported by a recent randomized controlled trial in which a 24 weeks statin monotherapy reduced the liver fat content quantified by MRI.^31^ Among those with NAFLD, statins may even be anti-fibrotic, however, this was only significant for a rather lenient definition of fibrosis using 7.0 kPa as threshold (which increased power). This anti-fibrotic property of statins has been shown in various experimental NASH models in which statins can inhibit the paracrine signalling between hepatocytes and hepatic stellate cells resulting in deactivation of these stellate cells, which in turn inactivates fibrogenesis.^32^^,^^33^

In addition, among biopsy-proven NAFLD patients, we found that statins were inversely associated with NASH (OR: 0.55). This adds to the evidence that statins may be hepatoprotective, in line with previous findings.^3^^,^^25^^,^^26^ However, no benefit of statins on fibrosis was observed in this cohort. This finding was unexpected, given the beneficial association between statins and NASH which is a major predictor for fibrosis.^34^ This may be explained by the relatively early stage of NAFLD disease, as supported by the younger population in the PERSONS cohort with better metabolic health, resulting in a lower prevalence of fibrosis than other biopsy-proven NAFLD patient cohorts.^35^ Moreover, only 14 out of 73 NAFLD patients had fibrosis while using statins, therefore, this part of the study may be underpowered.

To account for different outcomes across available studies as well as ours and to increase statistical power, level of evidence and generalizability of results, we performed a meta-analysis. Statins seem to have a preventative effect on the presence of NAFLD, although this did not reach statistical significance (OR: 0.69 95% CI: 0.46–1.01), and further validation is warranted. Furthermore, the pooled odds ratios indicated a significant protective effect of statin use on the presence of NASH (OR: 0.59) and fibrosis (OR: 0.48).

Interestingly, previous studies have shown larger effects on the development or progression of NAFLD for higher dosage and longer duration of statin use.^3^^,^^26^^,^^28^ This dose-dependent effect in itself supports the efficacy of statins in NAFLD. Besides the evidence from cross-sectional studies, similar protective effects of statin use were observed in longitudinal data. For example, in a meta-analysis the risk for hepatocellular carcinoma and mortality was lower among biopsy-proven NAFLD patients using statins, further highlighting the potential benefits of statins in NAFLD disease management.^36^

In addition to the clear benefits of statins on cardiovascular outcomes, there is now emerging evidence indicating that statin use is hepatoprotective. Therefore, among individuals with NAFLD, statin treatment—which is safe and inexpensive—might be indicated regardless of dyslipidemia to reduce the risk of advanced liver disease. Currently 40–50% of patients with NAFLD do not receive statin treatment, while it is indicated.^37^, ^38^, ^39^ Therefore, even prescribing statins according to recent guidelines^40^ might reduce the disease burden of NAFLD, as physicians involved in the multidisciplinary treatment of NAFLD should be aware of the potential hepatoprotective effects of statins. However, additional research is required on whether statins are effective in preventing NAFLD among those without an indication.

To illuminate the protective findings on liver health and demonstrate causality, we explored statins' direct anti-lipid properties in 3D cultured human liver organoids. Interestingly, we found that the high statin-concentration of 10 μM could significantly inhibit the number of induced lipid droplets, while on the other hand, the average size of remaining lipid droplets increased. Although this seems counterintuitive, this indicates a stronger inhibitory effect of statins on smaller lipid droplets than on larger ones. Whether prolonged exposure to statin treatment also results in the inhibition of these larger lipid droplets needs to be investigated in further studies. Last, while most in vitro experiments have used statin in the concentration of 1–50 μM, the reported serum concentrations in humans are much lower.^41^ Therefore, the experimental concentration of 10 μM may not reflect clinical practice. Nonetheless, a short experimental exposure to a high concentration of statins may be a proxy for chronic use of statins. This might explain that in several cohort studies the hepatoprotective effects were only relevant after six months of treatment^3^ and were stronger with higher cumulative dosage.^28^ This direct anti-steatosis effect might be explained by downregulating LDL, activating peroxisome proliferator-activated receptor alpha (PPARα) alongside increased β-oxidation, but further research is warranted to identify involved pathways.^42^^,^^43^

Last, we investigated the possible anti-inflammatory properties in monocytes differentiated macrophages, since macrophages have a crucial role in NASH.^44^ We demonstrated that the rather low concentration of 0.1 μM already downregulated the expression of inflammatory genes in macrophages such as CXCL10, IL-6, IL-8 IL-12, IL-1beta and IFN-gamma. Other studies have shown as well that statins may exert anti-inflammatory effects by inhibiting RhoA/Rho-kinase, a small GTPase that induces oxidative stress.^45^ Supporting our experimental findings, pooled data indicated that several inflammatory markers in serum of patients with metabolic syndrome were significantly decreased (e.g. CRP, IL-6 and IL-1).^46^ Furthermore, mRNA expression of IL-6 is increased in animal models of NASH.^44^ Interestingly, our study showed a decrease in expression of these inflammatory genes by statins. These anti-inflammatory properties might partially contribute to the hepatoprotective effect of statins.

The following limitations need to be mentioned. First, this study had a cross-sectional design and there was no data on the duration of statin therapy as well as treatment compliance, which limits the ability to investigate causality. Nonetheless, the additional experimental results show potential mechanisms that support causality. Second, our meta-analysis was based on only 3–7 studies, which did not allow for subgroup analysis and assessment of publication bias. Moreover, in the analysis concerning NAFLD and fibrosis there was substantial heterogeneity, particularly driven by a specific study,^28^ as this study used FLI and BARD score to define NAFLD and fibrosis. However, in excessive influence analysis, results were consistent after excluding this specific study indicating its limited impact on our conclusions. Third, the beneficial effect of statins may be partially explained by reverse epidemiology as there may be historical reluctance in prescribing statins to those with liver disease. However, under-prescription of preventive medicine is a general concern ranging from 22 to 70%,^47^ similar to the reported 40–50% under-prescription of statins in NAFLD and NASH patients.^37^, ^38^, ^39^ Therefore, the impact of this phenomenon on our results is limited. Although we have provided an experimental proof-of-concept regarding statins’ possible mechanisms-of-action, further in depth investigation in additional experimental models are warranted.

In summary, pooled results demonstrated that statin use was associated with lower odds of NASH and fibrosis. Moreover, statin use might also prevent NAFLD in patients with metabolic dysfunction. In our experimental models, statins inhibited lipid synthesis and downregulated the expression of pro-inflammatory cytokines, which may partially explain the clinical benefits. This emerging evidence for the hepatoprotective properties of statins should be considered in the disease management of NAFLD.

## Contributors

IA and LvK conceived the study and wrote the manuscript. IA, LvK, and HZ performed statistical analysis, which was approved and interpreted by MZ, RdK, and QP. YA, MG, LvL, HJ, and MP critically revised the manuscript for important intellectual content. PL, KL, and ML provided input on design of experimental part. IA, LvK, HZ, and QP have accessed and verified the data. All authors reviewed and approved the final manuscript.

## Data sharing statement

Data can be obtained upon reasonable request. Requests should be directed towards i.ayada@erasmusmc.nl and secretariat.epi@erasmusmc.nl. Requests regarding the PERSONS cohort should be addressed to zhanghuai@wmu.edu.cn.

## Declaration of interests

RdK received grants from Echosens, Abbvie, Gilead and Inventiva and received consulting fees from Abbvie, Gilead and Echosens. HJ received grants and consulting fees from Gilead, Janssen, GLaxoSmithKLine, Roche and Vir Biotechnology Inc. HJ received consulting fees from Eiger, Antios, Aligos. MP received consulting fees from Pfizer and Walvax Inc. The remaining authors reported no relevant conflicts.
