# Supplementary material for: Dissecting the multifaceted impact of statin use on fatty liver disease: A multidimensional study
Source: eBioMedicine. 2022 Dec 8;87:104392. doi: 10.1016/j.ebiom.2022.104392 (PMC9758527; doi:10.1016/j.ebiom.2022.104392)
Supplement: Supplementary Material [file mmc1.pdf]

Dissecting the multifaceted impact of statin use on fatty liver disease: a multidimensional study

Ibrahim Ayada, Laurens A. van Kleef, Huai Zhang, Kuan Liu, Pengfei Li, Yasir J. Abozaid, Marla Lavrijsen, Harry L. A. Janssen, Luc J. W. van der Laan, Mohsen Ghanbari, Maikel P. Peppelenbosch, Ming-Hua Zheng, Robert J. de Knegt, Qiuwei Pan

SUPPLEMENTARY MATERIAL

Table of contents

Supplementary table 1 ……………………….. 2

Supplementary table 2 ……………………….. 3

Supplementary table 3 ……………………….. 4

Supplementary table 4 …………………………5

Supplementary figure 1 ……………………….. 6

Supplementary figure 2 ……………………….. 7

Supplementary figure 3 ……………………….. 8

Supplementary method …………………………9

References …………………………13

Supplementary table 1: exclusion criteria

	Rotterdam Study
**Eligible for inclusion**	**5.967**
NAFLD exclusion criteria	
Viral hepatitis	44
Excessive alcohol	780
Steatogenic drug use	98
Incomplete data	
Alcohol consumption	408
Dyslipidemia	54
Statin use	7
**Total participants excluded**	**1.391**
**Participants included**	**4.576**

Abbreviations: NAFLD, non-alcoholic fatty liver disease

Supplementary table 2: database search and outcomes

	With duplicates	Without duplicates
Embase.com	1482	1124
Medline ALL Ovid	415	415
Web of Science SCI-EXPANDED & SSCI	406	180
Cochrane CENTRAL Register of Trials	126	47
**Total**	**2429**	**1766**

Embase.com

('hydroxymethylglutaryl coenzyme A reductase inhibitor'/exp OR (((hydroxymethylglutaryl* OR hydroxyl-methyl-glutaryl* OR hmg) NEAR/3 (coenzyme-A OR coa) NEAR/3 (inhibitor*)) OR statin* OR atorvastatin* OR bervastatin* OR cerivastatin* OR compactin* OR crilvastatin* OR dalvastatin* OR fluindostatin* OR glenvastatin* OR lovastatin* OR pitavastatin* OR pravastatin* OR rosuvastatin* OR simvastatin* OR tenivastatin*):ab,ti) AND ('nonalcoholic fatty liver'/exp OR (((nonalcohol* OR non-alcohol*) NEAR/3 (fatty-liver* OR hepatosteatos* OR steatohepatit*)) OR ((nonalcohol* OR non-alcohol*) NEAR/3 (liver* OR hepat*) NEAR/3 (steatos*)) OR nafld OR nash):ab,ti) NOT [conference abstract]/lim AND [english]/lim NOT ([animals]/lim NOT [humans]/lim)

Medline ALL Ovid

(exp Hydroxymethylglutaryl-CoA Reductase Inhibitors / OR (((hydroxymethylglutaryl* OR hydroxyl-methyl-glutaryl* OR hmg) ADJ3 (coenzyme-A OR coa) ADJ3 (inhibitor*)) OR statin* OR atorvastatin* OR bervastatin* OR cerivastatin* OR compactin* OR crilvastatin* OR dalvastatin* OR fluindostatin* OR glenvastatin* OR lovastatin* OR pitavastatin* OR pravastatin* OR rosuvastatin* OR simvastatin* OR tenivastatin*).ab,ti.) AND (Non-alcoholic Fatty Liver Disease / OR (((nonalcohol* OR non-alcohol*) ADJ3 (fatty-liver* OR hepatosteatos* OR steatohepatit*)) OR ((nonalcohol* OR non-alcohol*) ADJ3 (liver* OR hepat*) ADJ3 (steatos*)) OR nafld OR nash).ab,ti.) AND english.la. NOT (exp animals/ NOT humans/)

Web of Science SCI-EXPANDED & SSCI

TS=(((((hydroxymethylglutaryl* OR hydroxyl-methyl-glutaryl* OR hmg) NEAR/2 (coenzyme-A OR coa) NEAR/2 (inhibitor*)) OR statin* OR atorvastatin* OR bervastatin* OR cerivastatin* OR compactin* OR crilvastatin* OR dalvastatin* OR fluindostatin* OR glenvastatin* OR lovastatin* OR pitavastatin* OR pravastatin* OR rosuvastatin* OR simvastatin* OR tenivastatin*)) AND ((((nonalcohol* OR non-alcohol*) NEAR/2 (fatty-liver* OR hepatosteatos* OR steatohepatit*)) OR ((nonalcohol* OR non-alcohol*) NEAR/2 (liver* OR hepat*) NEAR/2 (steatos*)) OR nafld OR nash))) AND DT=(article) AND LA=(english)

Cochrane CENTRAL Register of Trials

((((hydroxymethylglutaryl* OR hydroxyl-methyl-glutaryl* OR hmg) NEAR/3 (coenzyme-A OR coa) NEAR/3 (inhibitor*)) OR statin* OR atorvastatin* OR bervastatin* OR cerivastatin* OR compactin* OR crilvastatin* OR dalvastatin* OR fluindostatin* OR glenvastatin* OR lovastatin* OR pitavastatin* OR pravastatin* OR rosuvastatin* OR simvastatin* OR tenivastatin*):ab,ti) AND ((((nonalcohol* OR non-alcohol*) NEAR/3 (fatty-liver* OR hepatosteatos* OR steatohepatit*)) OR ((nonalcohol* OR non-alcohol*) NEAR/3 (liver* OR hepat*) NEAR/3 (steatos*)) OR nafld OR nash):ab,ti)

Supplementary table 3: Overview of studies reporting on statin use and NAFLD, NASH or fibrosis among individuals with metabolic dysfunction

Author	Study characteristics			Patients characteristics				Hepatic assessment		
	Population	Country	n	Age(y)	Male(%)	BMI	Diabetes(%)	NAFLD	NASH	Fibrosis
Boon-Bee Goh et al., 2014	Diabetes	USA	220	52	31	37	100	Biopsy	Biopsy	Biopsy
Oni et al., 2014	Metabolic syndrome	Brazil	1.277	43*	79*	25*	N.E.	US	–	–
Dongiovanni et al., 2015	NASH + diabetes	Europe (multi-center)	1.059	42	52	34.2	27	Biopsy	Biopsy	Biopsy
Nascimbeni et al., 2016	Gastric bypass	Italy	346	53	40	42	74	Biopsy	Biopsy	Biopsy
Ciardullo et al., 2021	Diabetes	USA	744	61	52.6	33.3	100	CAP ≥274	–	LSM ≥9.7
Lee et al., 2021	Diabetes	Korea	60.918	41*	71*	24.4*	100	FLI ≥60	–	BARD ≥2.0
Rotterdam Study	Dyslipidemia or NAFLD**	Netherlands	2.408	71	45	28.5	24	US	–	LSM ≥8.0
PERSONS Cohort	NAFLD	China	569	42	72	26.6	25	Biopsy	Biopsy	Biopsy

Abbreviations: NAFLD, non-alcoholic fatty liver disease; NASH, non-alcoholic steatohepatitis; BMI, body mass index (kg/m^2); NE, not extractable.
*based on whole study population rather than the included population with metabolic dysfunction in the meta-analysis
**Participants with dyslipidemia were assessed for the presence of NAFLD, and participants with NAFLD for the risk of fibrosis.

Supplementary Table 4: Quality assessment of studies included in meta-analysis

Authors	Year	Selection	Comparability	Outcome	Total
Ciardullo et al.[1]	2021	★★★★	★	★★★	8/9
Dongiovanni et al.[1]	2015	★★★★	★★	★★★	9/9
Goh et al.[1]	2014	★★★★	★	★	7/9
Lee et al.[2]	2021	★★★	★★	★★★	8/9
Nascimbeni et al.[1]	2016	★★★	★★	★★★	8/9
Oni et al.[1]	2014	★★★	★★	★★	7/9

[1] The Newcastle-Ottawa quality assessment scale for cross-sectional cohort studies was used
[2] The Newcastle-Ottawa quality assessment scale for case-control studies was used

A

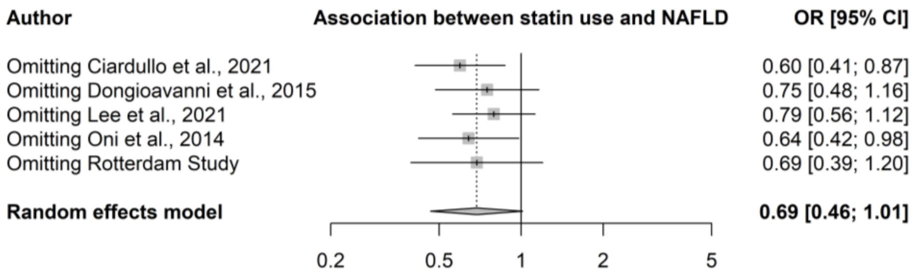

B

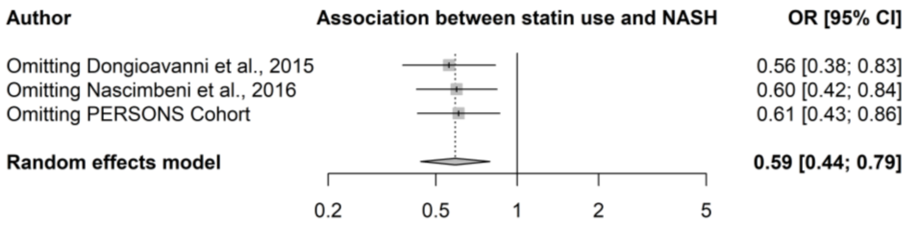

C

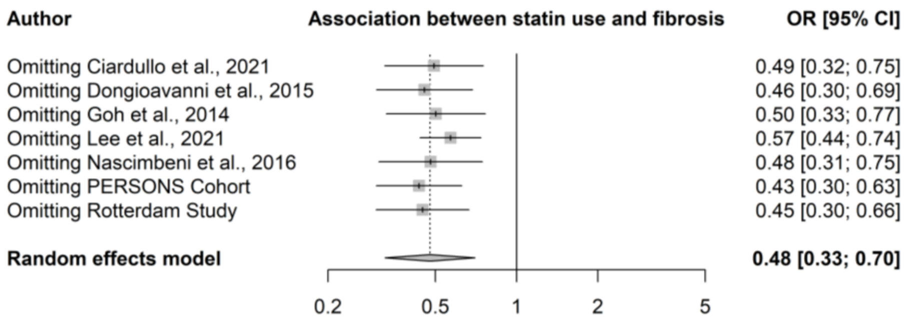

Supplementary figure 1: Excessive influence analysis in which we omitted one study at a time for the associations between statin use and NAFLD (A), NASH (B) and fibrosis (C).

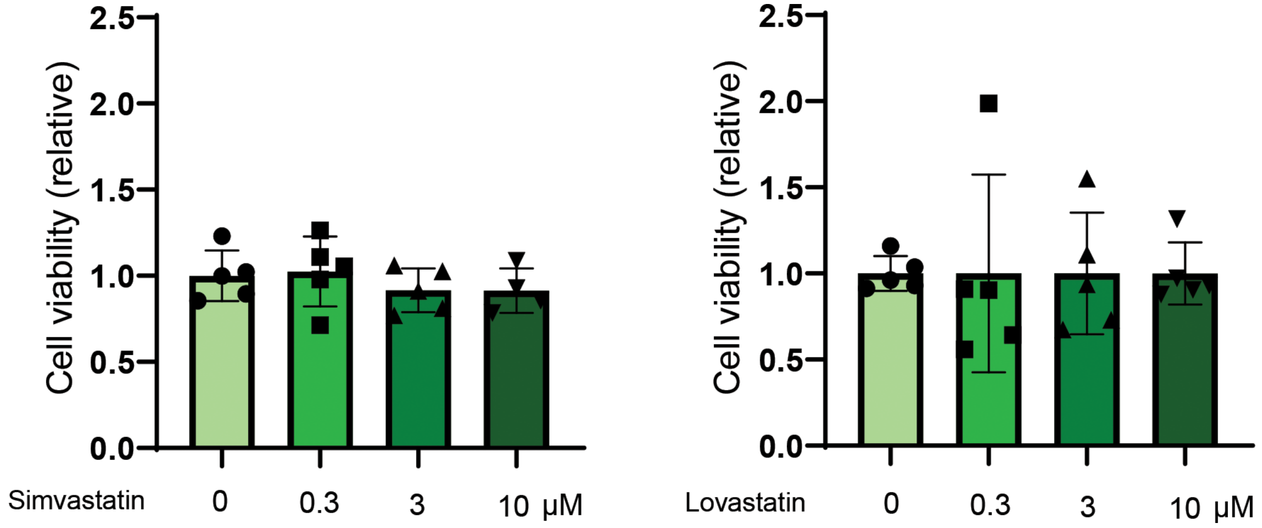

Supplementary figure 2: Quantification of organoids viability by AlamarBlue assay. Organoids were exposed to LPO and treated with increased concentrations of simvastatin and lovastatin for 96 hours or without treatment as control, (n = 5).

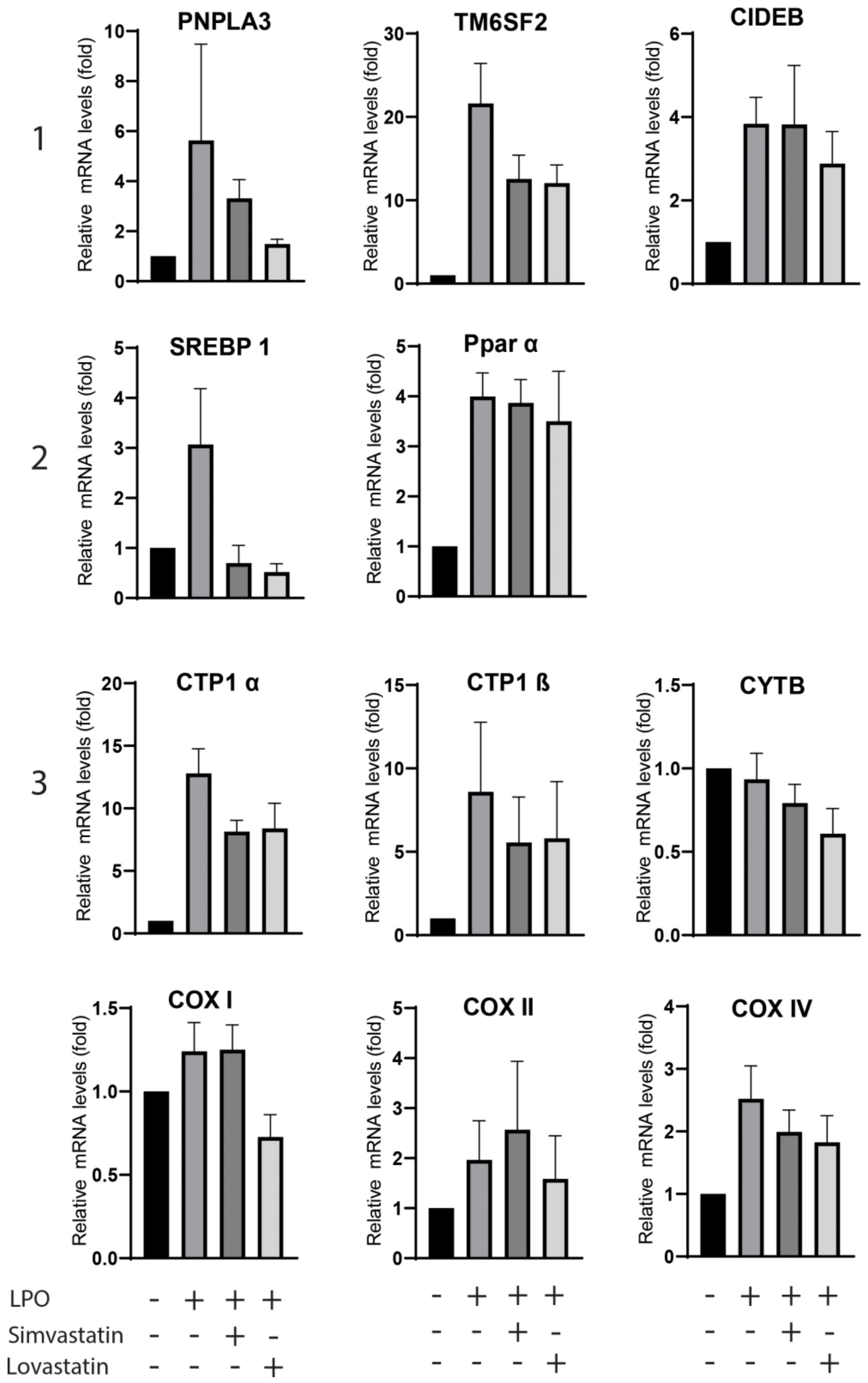

Supplementary figure 3: The effects of statin treatment on expression of genes involved in steatosis (panel 1), fatty acid metabolism (panel 2) and mitochondrial function and morphology (panel 3). Human liver organoids were exposed to LPO medium and LPO with simvastatin or lovastatin for 96 hours. Relative gene expression is represented as CTR (untreated, normal liver expansion medium) and as treated with statins in the concertation of 10 µM, (n = 5).

Supplementary methods

Study selection

Articles were screened and included by I.A. if they met the following criteria: adult population with data available regarding the presence of NAFLD and usage of statins, conducted from cohort studies from selected patients or populations. Exclusion criteria were non-human studies, duplicates, non-original data or abstracts. Investigator I.A. screened titles and abstracts and subsequently full texts of potentially eligible articles found by the search strategy. The Preferred Reporting Items for Systematic Reviews and Meta-Analyses (PRISMA) flowchart was used to create an overview of the data screening process and the PRISMA NMA checklist (supplementary figure 1) as guidance for reporting on all the required aspects of a meta-analysis.

Data extraction and quality assessment

Data extraction was performed independently by two authors (I.A. and L.v.K). The Newcastle-Ottawa quality assessment scale for for cross-sectional studies were used for quality assessment, which can be found in **supplementary table 4**. Discrepancies were resolved by mutual discussion among authors (I.A. and L.v.K).

Liver organoids culture

Organoids capture some of the key multicellular, anatomical and even functional hallmarks of real organs, thus have an advantage compared to classical cell lines. Studies have demonstrated that organoids can be used to model organ development and disease. Primary organoids are cultured from tissue stem cells in 3D structure, thus retaining characteristics of the tissue of origin. Therefore, for this model human intrahepatic cholangiocyte organoids (ICO) were used.

Organoids were cultured in matrigel with Advanced DMEM/F12 (Life Technologies, cat.no.12634-010), adding 1 M HEPEs (Lonza, cat. no. 17-737E), ultraglutamine (Lonza, cat. no. BE17-605E/U1) and penstrep as the basic culture medium, supplied with 1:50 B27 supplement (minus vitamin A), 1:100 N2 supplement, 1 mM N-acetylcysteine, 10 mM nicotinamide, 50 ng/ml EGF, 100n g/ml FGF-10, 50 ng/ml HGF, 5 μM A83-01, 10 μM forskolin, 10 nM gastrin and 10% R-spondin1 (produced by 293T-H-RspoI-Fc cell line). [1] The organoids were cultured for approximately one week in which the medium was refreshed every 72 hours. When the appropriate size was reached, steatosis mimicking condition were applied after adding sodium lactate, sodium pyruvate and octanoic acid (LPO). Statin treatment was initiated for 96 hours. The use of human liver tissues for research purposes was approved by the Medical Ethical Council of Erasmus MC and informed consent was given (MEC-2014-060).

Staining of lipid droplets

Firstly, induction of lipid synthesis was initiated by adding lactate, pyruvate and octanoic acid to the organoid expansion medium.[2, 3] Lactate and pyruvate are physiological derivates of both gluconeogenesis and de novo lipogenesis. Octanoic acid is a medium chain fatty acid which induces triglycerides accumulation. Lipids and nuclei were subsequently stained with AdipoRed (Lonza, cat.no.PT-7009) and Hoechst 33342 (Life Technologies, cat.no.H3570) respectively after disrupting the matrigel construction followed by spinning down in order to remove excess matrigel. The stained organoids were then incubated for 20 minutes in a dark incubator at 37 degrees Celsius. Next, we washed and spun down once with 1 X PBS, followed adding the organoids including anti-fading medium on glass slides. Images were captured by confocal microscope Zeiss LSM510meta and Leica SP5, and quantified with ImageJ software. Analysis

was performed by splitting the individual color channel for lipids and threshold converting to 8-bit. The acquired images were measured with particles and their surface areas in pixels, then converted to square micrometers for lipids areas.[2]

Culturing of THP1-cells

Human monocytic cell line (THP-1) was cultured in RPMI 1640 medium (ThermoFisherScientific, Waltham, MA, USA), complemented with 10% (v/v) inactivated fetal bovine serum (FBS) with 100 IU/mL of penicillin and 100 mg/mL of streptomycin. For macrophage differentiation, THP-1 cells were treated with 20 ng/mL of phorbol 12-myristate 13-acetate (PMA) at 37°C for 48 hours. Then, cells were cultured for another 24 hours in RPMI 1640 medium without PMA. Treatment with statins was sustained for 24 hours followed by qRT-PCT. Gene expression of cytokines including CXCL10, TNF alpha, IL8, IL18, IL6, IL12, IFN gamma and IL1beta were quantified by qRT-PCR. GAPDH was used as housekeeping gene for normalization of gene expression.

Quantification of gene expression

Total RNA was isolated using the Macherey-Nagel NucleoSpin RNA II Kit (Marchery-Nagel GMBH & Co, Düren, Germany) and subsequently quantified by Nanodrop ND-1000 (Wilmington, DE, USA). RNA expression levels were quantified by SYBR Green–based qRT-PCR (Applied Biosystems SYBR Green PCR Master Mix; Thermo Fisher Scientific Life Sciences) with the StepOnePlus System (Thermo Fisher Scientific Life Sciences). Glyceraldehyde 3-phosphate dehydrogenase (GAPDH) was used as housekeeping gene in all qPCR experiments involved in this study. Relative gene expression was normalized to GAPDH using the formula $2^{-\Delta\Delta CT}$ ($\Delta\Delta CT = \Delta CTsample - \Delta CTcontrol$).

AlamarBlue assay

The supernatant (organoids expansion medium) in which the organoids were cultured was discarded first. Second, the organoids were incubated with AlamarBlue solution (Invitrogen, DAL 1100) which was 20 fold diluted in organoid expansion medium. After an incubation time of 120 minutes at 37 °C, the diluted AlamarBlue solution was removed and transferred to a 96 wells plate in order to measure absorbance by using fluorescence plate reader (CytoFluor Series 4000, PerSeptive Biosystems) at the excitation level of 530/25 nm and emission level of 590/35 nm

References

[1] Broutier L, Andersson-Rolf A, Hindley CJ, Boj SF, Clevers H, Koo B-K, et al. Culture and establishment of self-renewing human and mouse adult liver and pancreas 3D organoids and their genetic manipulation. Nature Protocols 2016;11:1724-1743.

[2] Wang L, Li M, Yu B, Shi S, Liu J, Zhang R, et al. Recapitulating lipid accumulation and related metabolic dysregulation in human liver-derived organoids. J Mol Med (Berl) 2022.

[3] Lyall MJ, Cartier J, Thomson JP, Cameron K, Meseguer-Ripolles J, O'Duibhir E, et al. Modelling non-alcoholic fatty liver disease in human hepatocyte-like cells. Philos Trans R Soc Lond B Biol Sci 2018;373.
